# Operative Dentistry as Applied to Deciduous Teeth

**Published:** 1887-11

**Authors:** Wm. N. Morrison

**Affiliations:** St. Louis, Mo.


ARTICLE VI.

### OPERATIVE DENTISTRY AS APPLIED TO DECIDUOUS TEETH.

BY DR. WM. N. MORRISON, ST. LOUIS, MO.

The text-books, and notably Tomes and Harris, are very meagre in their treatment of deciduous teeth. The journals are more advanced, and through them Profs. J. D. White and J. H. McQuillen have battled nobly for their retention. In the "American System of Dentistry," the deciduous teeth are highly esteemed, and their rational treatment plainly set forth. It says:

"Well constituted amalgam and tin foil are the most durable; and on the whole make the most satisfactory materials to use. As little children cannot employ the means to cleanse the teeth which adults use, the adjoining surfaces of fillings should be brought into contact at the risk of the recurrence of decay. If spaces are permitted to exist, they become packed with ingesta, which leads to soreness of the gums, disuse of the teeth, and the early recurrence of decay."

The profession is still far in advance of both the text-books and the journals, and to the "Grand Old Man," Dr. W. H. Atkinson, are we most indebted for the progressive ideas in regard to the preservation of these teeth. At no period in our professional history have the deciduous teeth received so much intelligent preservative care as they do to-day. And yet there is much that could be done in this direction that is cast aside as a mere trifling matter, and the tooth extracted for want of time to treat it, or to give the parent a few hours more sleep.

In my earliest recollections of discussions in the St. Louis Dental Society, more than a quarter of a century ago, a prominent member, in the most tragic manner, asserted that if it were possible, with a keen scimiter to excise the entire deciduous process and teeth from the jaw, no unfavorable effect could be observed in the permanent teeth when the individual had arrived at maturity. The floor was very eagerly occupied in debate by Leslie, Dunham, Peebles, Comstock, Barron and Forbes, who are now delegates to that Society from which members never return.

When around the festive board, at the close of the meeting, it was explained that the tragic statement was not meant to be taken in its literal sense, but was intended to waken the members to a discuscion of the subject, such as its great importance demanded. It certainly had that effect and only intensified the general jollification which followed.

It should be our aim to save all of the crowns in their natural shape and position, and by a proper degree of care keep them thoroughly cleansed, thereby preventing their decay.

Simple, regular-shaped cavities can be filled with gold, in cylindrical form, preferably; and it would be an exceptional case where retaining pits would be admissable. Continuous malleting, or much force, should not be used upon these delicate organs, separated as they are by so slight a framework from the more delicate permanent ones in their impressionable formative stage.

Amalgam has a wider range of usefulness, and can be used for simple cavities; and for the larger and more complicated it has no superior.

I would not excavate heroically, as too much cutting and excavating only endangers the life of the pulp. Do not gratify that desire to take away one more layer, just to see how close you can go to the pulp and have it live under the filling; leave the soft denture over the pulp tissues, securing slight undercuts only, merely enough to prevent the fillings from being lifted from their beds. When pulps are exposed, they should be capped with gutta percha dissolved in chloroform, or phosphate, where there is the slightest hope of their remaining alive; but when they are dead, I remove all the tissue from the pulp chamber and canals as thoroughly as possible, and disinfect the canal with a little wood creosote to arrest hemorrhage and purify the canal, and then fill the roots and pulp chamber with the gutta percha mixture, and the external cavity with amalgam, being careful to polish the amalgam fillings at a subsequent sitting.

The phosphates and gutta percha are of too uncertain action, and too temporary in character, for even deciduous teeth, gutta percha being acted upon by the secretions of the mouth and swelling out of the cavity, and the phosphates being too readily washed away.

About the time the centrals are erupted, there is considerable pressure on the approximal surfaces, and when decay at those points is neglected, the surfaces seem to drop quite into each other. Now, in the last instance, I wedge them open with cotton for a few days, and if the cavities are of such shape that individual fillings cannot be made so as to retain themselves in each tooth separately, I would block the entire space full, reaching from the undercut of the enamel of one crown across to the undercut in the other tooth, leaving the whole mass of filling bridged from one tooth to the other, vastly improving the power of mastication, keeping the arch braced to assist in the development of the secocd teeth, which is taking place below.

I rarely extract the devitalized roots, but fill them and grind them smooth, and but little short of occlusion.

Educate the parents, as well as the children, and then there will not be the exclamation when they bring the sixth-year molar, decayed almost beyond recognition, "I thought it was a baby tooth!"

Too much sweets and trashy diet are the cause of such imperfectly organized teeth, and the approximal pressure on the vitreous enamel, surrounded by such secretions, greatly accelerates their decay. Teach patients how to forcibly oxygenate their blood with the purest out door air they can get; filling the lungs to their fullest extent, holding it a moment, and then contracting the chest muscles upon them, thus forcing the air into the remote territory which is not used on ordinary occasions. Use boiled water to drink, whether hydrant, cistern or well, purifiying it by bringing it to the boiling point, thus destroying much organic life which could not be other than harmful. Use sulphur as a dentifrice, to destroy any parasitical life that may escape. So, with nature's bountiful gifts—pure air, pure water, and a plain diet and plenty of sunlight—a magnificient physique will be built up and the race improved. By this line of treatment, in a few generations, irregularities of the permanent teeth would be of very rare occurrence.

The "Hearth and Home" is doing a noble missionary work towards educating the public in the care and preservation of the teeth.

Let all of our efforts upon these teeth be towards their preservation to the very last hour the individual can make any use of them; and let it be our aim to impress upon parents the necessity of proper care and attention on their part. The value of deciduous teeth has never been fully appreciated by parents, and too little care bestowed upon them. I regard them as being just as important in their sphere as permanent teeth are in theirs.

I beg leave to submit for your examination a few casts to illustrate the line of treatment.

In a paper on "The Treatment of Deciduous Teeth," read before the St. Louis Dental Society several years ago, I set forth at some length my method of treating deciduous teeth; by an oversight this article was not published at the time, but appears in the May number of the "Archives of Dentistry." I most heartily commend it to your careful consideration.—*Transactions of the Illinois State Dental Society.*

